# Mobile Cloud-Computing-Based Healthcare Service by Noncontact ECG Monitoring

**DOI:** 10.3390/s131216451

**Published:** 2013-12-02

**Authors:** Ee-May Fong, Wan-Young Chung

**Affiliations:** Department of Electronic Engineering, Graduate School, Pukyong National University, Busan 608-737, Korea; E-Mail: eemay88@hotmail.com

**Keywords:** ECG monitoring, healthcare cloud service, personalized healthcare assistant, mobile device

## Abstract

Noncontact electrocardiogram (ECG) measurement technique has gained popularity these days owing to its noninvasive features and convenience in daily life use. This paper presents mobile cloud computing for a healthcare system where a noncontact ECG measurement method is employed to capture biomedical signals from users. Healthcare service is provided to continuously collect biomedical signals from multiple locations. To observe and analyze the ECG signals in real time, a mobile device is used as a mobile monitoring terminal. In addition, a personalized healthcare assistant is installed on the mobile device; several healthcare features such as health status summaries, medication QR code scanning, and reminders are integrated into the mobile application. Health data are being synchronized into the healthcare cloud computing service (Web server system and Web server dataset) to ensure a seamless healthcare monitoring system and anytime and anywhere coverage of network connection is available. Together with a Web page application, medical data are easily accessed by medical professionals or family members. Web page performance evaluation was conducted to ensure minimal Web server latency. The system demonstrates better availability of off-site and up-to-the-minute patient data, which can help detect health problems early and keep elderly patients out of the emergency room, thus providing a better and more comprehensive healthcare cloud computing service.

## 1. Introduction

Recently, the intrusion of cardiovascular disease into society has increased to the point where it has become the leading cause of death worldwide. According to the 2012 World Health Organization (WHO) Statistics report [1], cardiovascular diseases accounted for the largest proportion of noncommunicable disease deaths, which is 48%. WHO has also gathered a series of graphs based on global health data to illustrate the 10 leading causes of death by broad income groups such as in low-, middle-, and high-income countries. Table 1 shows the top-three leading causes of death by broad income groups [2]. Heart disease and stroke and other cerebrovascular disease represent the top-two causes of death in the middle- and high-income nations.

Heart-attack patients have been proven to have a sudden death rate of four to six times that of the general population [3]. These critical issues have raised public concern regarding the need for a cardiovascular healthcare system, and a growing demand for better quality of life has arisen. Furthermore, the stress suffered by the people, especially the working community, in this progressive society, often leads to a higher rate of patients suffering from hypertension, orthostasis and vascular disease. Continuous health monitoring by using heart rate variability (HRV) parameters is one of the main purposes for ECG measurements [4]. With a continuous health monitoring system, a series of HRV parameters can be collected, the cardiovascular function continuously monitored and therefore an essential overall health evaluation can be performed for early detection and treatment of cardiovascular anomalies, particularly in high-risk patients. However, the overall global healthcare costs increase when the demand for medical services increases, and healthcare financial cost increases have become a huge challenge to governments, health organizations, and the public. Report shows that 16% of the GDP in the USA was spent on healthcare in 2009 [5]. Consequently, many people are unwilling to seek medical help until their conditions have worsened.

With the development of technologies such as mobile computing, wireless sensor networks, and Web server cloud computing, the required medical care and medical consultation can be provided easily and remotely. Advanced information and communication technologies in medical healthcare services can not only reduce the manpower requirements and medical healthcare costs, but also achieve real-time medical care service and provide advanced care to remote locations. Mobile healthcare systems enable remote healthcare monitoring, and the Web server cloud computing system enables remote data collection. To remotely and continuously monitor the electrocardiograms (ECGs) from multiple locations, biomedical data are uploaded from mobile devices and shared on the Internet.

This system utilizes mobile devices to display and transmit physiological signals such as the ECG signal and heart beat rate. Then, the health information is collected, synchronized, and shared instantaneously over the Web server through Wi-Fi or a data network, as any heart attack or heart disease must be diagnosed immediately. The raw health data are stored in cloud storage to enable professional medical personnel to access and analyze the monitoring results, providing appropriate care services. Using the embedded Web server and dynamic Web page technology, the health status is displayed on a Web page; family members can check the medical records at any convenient time.

In this study, we aim to develop a mobile cloud-based healthcare system using a capacitive-coupled ECG monitoring system. This system comprises the following aspects: (1) nonintrusive real-time health status monitoring; (2) analysis of the biomedical signals obtained from sensors by a decision-making module for immediate health status checking; (3) implementation of mobile healthcare system with multiple-function life-assistant services in mobile device; and (4) Web server cloud-based service for synchronization of health status for seamless and continuous remote health tracking.

## 2. Background and Related Works

An ECG signal is an electrical signal generated by the heart's beating, which can be used as an important diagnostic tool to examine the heart function. Researchers have carried out experiments to investigate the effects of the resting heart rate (HR) on the health status of participants. The resting HR is defined as the number of heart beats per minute (bpm) measured under a condition of complete rest. It reveals the pumping efficiency of the heart and ranges between 60 and 100 bpm for adults. A value closer to the lower limit of this range qualifies as a good resting HR. A resting HR beyond the normal range is considered an indication of cardiac arrhythmia if accompanied with dizziness, chest pain, and short breaths, which may be a result of heart damage due to aging, electrolyte imbalance, high blood pressure, and cardiac disorder.

The Netfit news from the UK [6] indicated that a high resting HR, which is 15%–25% beat higher than a person's normal HR, or a large fluctuation in a human's resting HR reading is an early indication that training is too hard. In addition, Legeai *et al.* [7] found that participants with higher resting HR (79 bpm or above) had an 85% increased risk of cardiovascular and noncardiovascular mortality compared with those with a lower resting HR (62 bpm or lower). In one study published in the *American Journal of Hypertension* [8], researchers found that adults who have resting HRs above 80 bpm are more likely to become obese and develop diabetes after two decades. Thus, the recording of the ECG signal or HR over long periods of time during everyday activity could be useful in managing individuals with chronic health disorders. Moreover, it is important in assessing the effects of treatment at home and is potentially beneficial in observing the deviations in health status from the norm at an early stage or in automatically alerting paramedics in emergency cases.

**Figure 1.**
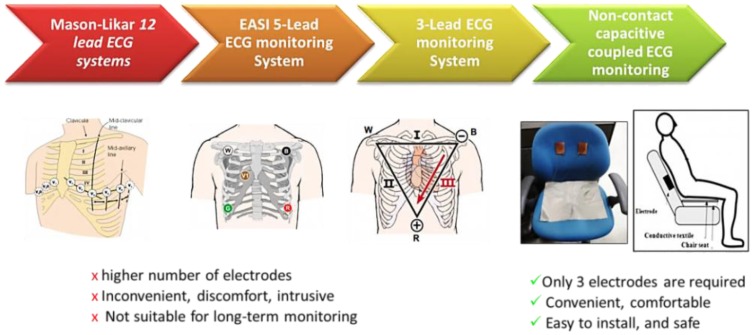
Comparison between the conventional and noncontact capacitive-coupled ECG measurement methods.

Figure 1 shows the comparison between the conventional and the noncontact ECG measurement techniques. In the conventional clinical ECG monitoring system, 12 or 15 Ag-AgCl electrodes (wet ECG) are affixed to specific parts of the chest, arms, hands, and legs. Although these conventional ECG measurements method gives more comprehensive information about the heart and achieves a better ECG signal quality, but the electrodes contain an electrolytic paste, are always required to maintain a reliable ohmic contact with the skin to provide a conducting medium for charge transfer between the electrodes and the body. Although this type of ECG provides good signal quality, it is inconvenient and may cause skin irritation and allergic reactions in long-term treatments because of the toxicological issues of the gels. Therefore, a wet ECG electrode system may be unsuitable for long-term ECG monitoring.

**Figure 2.**
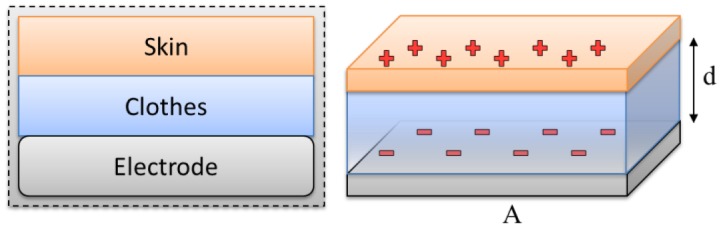
Body–electrode interface that forms the coupling capacitance.

In the capacitive-coupled noncontact method, no direct contact with the body is required. The electrode, together with the skin and the insulator, forms a parallel plate capacitance that conveys the signal from the body to the sensor, as shown in Figure 2. Thus, any electronic activity in the body will cause an electronic field to be formed over the isolating material of the capacitor plate. By measuring the field or the voltage induced by the field, the electronic body signals can be measured without direct galvanic contact with the body.

The capacitance formed between the body, insulator, and sensor electrodes can be generally described by Equation (1):

(1) $$C_{c}=\frac{\varepsilon_{0}\varepsilon_{r}A}{d}$$

This approach frees the user from inconvenience, such as battery replacement in the portable devices, and avoids any cumbersome electrodes or wires. This property allows the integration of a measurement system into everyday objects and allows continuous measurement, without placing constraints on the patients. Since its introduction by Lopez and Richardson [9], the application of a capacitively coupled ECG monitoring method has been extended to various environments. Lim *et al.* applied it into an office chair [10] and a bed [11]. Kato *et al.* performed electrocardiography using an incubator mattress system [12]. Leonhardt *et al.* proposed noncontact ECG monitoring in automobiles [13]. Jung *et al.* analyzed electrode positions for a highly sensitive driver monitoring system [14]. Schumm *et al.* demonstrated physiological monitoring from an airplane seat [15].

Currently, revolution in the development of smart devices and health-related applications is taking place; healthcare is entering an IT period with mobile and cloud computing technologies at the heart of the healthcare transformation. First, mobile healthcare can remotely monitor and analyze health status. Second, cloud-based technology allows health data to be synchronized and shared on a cloud computing system where more efficient processes and fast emergency response can be provided. Lee *et al.* [16,17] demonstrated smart-phone-based biosignal driver alertness monitoring whereas Hii *et al.* [18,19] presented healthcare solutions using Android-based mobile devices. Utilizing smart electronic devices for personal healthcare can reduce day-to-day healthcare costs by enabling healthcare professionals to have access to comprehensive, real-time patient data at the point of care or, indeed, anywhere with cellular network coverage. Therefore, in our system, advancements are being made toward a cheap and effective means of health monitoring.

## 3. System Design and Implementation

**Figure 3.**
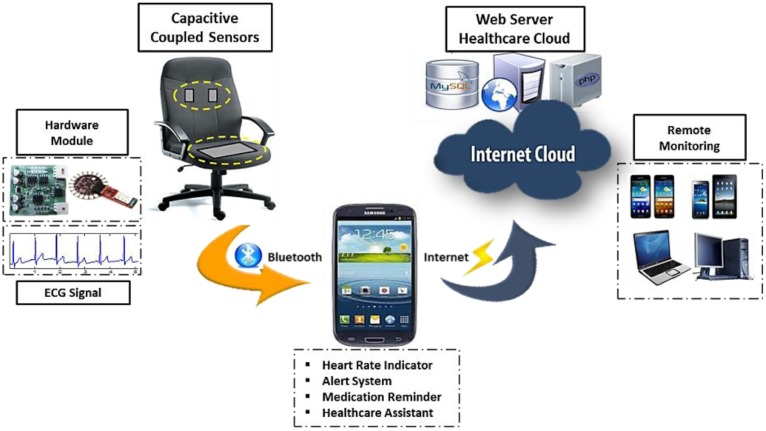
System design of mobile healthcare and cloud-based service for noncontact ECG monitoring system, which consists of a data acquisition module, a mobile system, and a cloud computing Web server module.

A noncontact ECG measurement system aims to continuously measure the biosignal on chairs. This approach can be applied on different chairs at multiple locations to continuously keep track of the health status of a patient, which is essential as a human spends much time on the chair every day. It may be an office chair when the user works in the office or he may be driving an automobile from one destination to another. More often, the elderly spends his day at a home sofa watching his favorite TV series, or the young ones may spend a few hours per day in their study room chair to study or work at home. The proposed system is mainly used for ECG monitoring with a mobile healthcare system and cloud-based service. Figure 3 shows the system design of the proposed real-time ECG monitoring system and health status analysis with a mobile device. Simultaneously, the mobile device acts as a healthcare assistant for the user. To continuously and seamlessly monitor the biosignal, a Web server healthcare cloud computing system is employed for health status sharing purposes.

### 3.1. Data Acquisition Module

**Figure 4.**
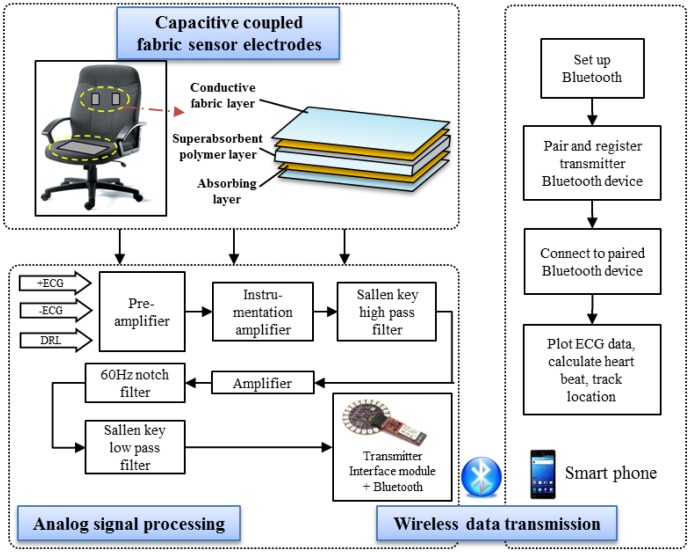
Flowchart of the data acquisition module.

The flowchart of the data acquisition module is shown in Figure 4. The hardware architecture includes the fabric sensor electrodes and the sensor module for analog signal processing. The software architecture includes Bluetooth communication between the transmitter (Arduino microcontroller and Bluetooth Mate) and receiver (mobile) devices.

The conductive fabric sensor electrodes are redesigned to ensure integrity of the sensor device and to measure clear and high-quality ECG signals. Then, the flow of data acquisition is followed by analog signal processing for signal filtering. Subsequently, the biosignal is transmitted via Bluetooth communication. At the receiver side, a smart phone plots the received ECG data. The heart beat rate is calculated, and the location is simultaneously tracked.

ECG monitoring using a human-machine-interface (HMI) is a current commercial solution in medical sector; however ECG HMI potential applications are limited [20]. Our research work presents improvement in sensors, embedded processing and heart beat detection algorithm. A hygroscopic fabric electrode has an embedded superabsorbent polymer layer is designed as sensor electrode to ensure a high humidity condition. It also ensures a strong coupling and to allow for the measurement of a stable, clear biomedical signal with higher QRS amplitude. Baseline wandering removal and signal filtering are implemented in analog signal processing. After considering the environment noise in the ECG recording, we designed an analog circuit for optimal performance. Our method operates through a pair of capacitively coupled electrodes installed at the back of a chair and a conductive textile installed on the seat for capacitive driven-right-leg (DRL) grounding. The DRL circuit was designed to suppress the interference caused by the finite common-mode rejection ratio of the instrument amplifiers. The capacitive electrodes are connected to an electronic circuit that includes high-input impedance amplifiers and band-pass filters to eliminate the analog noise. This included a low-pass filter for analog signal noise filtering and a high-pass filter for baseline wandering elimination. A notch filter is used to eliminate the power line noise of 60 Hz [21].

### 3.2. Mobile-Based Healthcare Service

Mobile devices are evolving at a rapid pace in the deployment of healthcare services. Our system is mainly based on real-time long-term health monitoring, catering to the demand of assisted living and health fitness information provider. Thus, the deployment of mobile devices into the mobile healthcare system focuses on several significant features for a medical healthcare system.

#### 3.2.1. Communication between Mobile Device and Web Server

**Figure 5.**
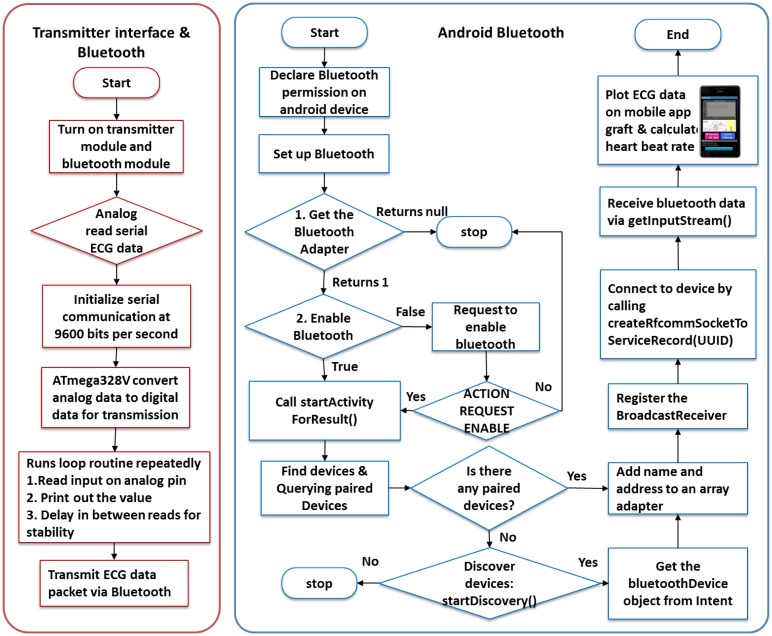
Bluetooth communication between the transmitter and mobile devices.

Bluetooth data transmission is applied into the system because Bluetooth facilities are available in numerous smart devices, including portable tablet devices, laptops, personal computers, and even smart TVs. Conceptually, Bluetooth is a mature and open wireless protocol operating in the 2.4-GHz band designed for a medium data rate that averages approximately 2 Mbps over a typical range between 10 and 50 m. Bluetooth communication between the transmitter and mobile devices is shown in Figure 5.

To transmit data, the proposed system uses a Lilypad Arduino (Mouser Electronics Inc., El Cajon, CA, USA), and the Bluetooth module connects to the output of the analog circuitry to read the analog serial data. Using its built-in analog-to-digital converter, the microcontroller ATmega328V digitizes the analog ECG data into data packets, which are sent continuously via Bluetooth. Smart devices with an Android-based Bluetooth application programming interface (API) allow the application to perform the following data reception tasks: scanning of the Bluetooth devices, querying the local Bluetooth adapter for paired Bluetooth devices, establishing radio frequency communication channels, connecting to the specified sockets of other devices, and transferring data to the other devices. On the mobile device, the Bluetooth APIs pair and connect to a particular Bluetooth device by calling for a specific universally unique identification number to ensure that a correct connection receives the Bluetooth data, as well as plot the ECG signals on a mobile application graph while calculating the HR and displaying it on the mobile application interface.

#### 3.2.2. Mobile Healthcare and Life Assistant

**Figure 6.**
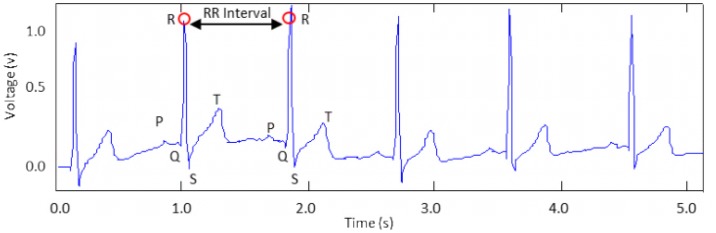
Peak detection of the ECG signal.

After the biosignal is measured, the HR is calculated by the Android mobile system, which is essential to monitor the heart beat rate and to send system alert if an abnormal HR is detected. Generally, HR refers to the rate of ventricular contractions. It is usually derived from the ECG by an algorithm that detects a single heart beat. The HR calculation can be easily done because each heart beat is accompanied in the ECG by a QRS complex. The ECG signal is filtered using the coefficient wavelet transform (CWT) with scale 1 and wavelet coiflet −1 to further remove unwanted noise such as baseline shift and motion artifacts. Next, the R peaks, which have the largest amplitudes, are detected by setting a threshold value. Figure 6 shows that the RR intervals of the ECG signal are detected. By measuring the consecutive intervals between heart beats (RR intervals), the HR can be easily calculated.

As shown in Equation (2), the inverse of the time difference between the normal heart beats gives the HR. HR is expressed in beats per minute (bpm) unit.

(2) $$HR(\text{bpm})=\frac{60}{RR\;\text{Intervals}\;(s)}$$

In the proposed system, the ECG signals are measured, and the HR is calculated by the mobile device in real time. The HR is shown on the mobile interface to allow users to easily view them. Whenever an abnormal HR is detected, that is, outside the range 60–100 bpm, the mobile device sends a notification or warning to alert the user. However, the location of the user is unknown. Implementing real-time location tracking is thus imperative so that when a medical emergency occurs, rescue and help can be provided immediately.

**Figure 7.**
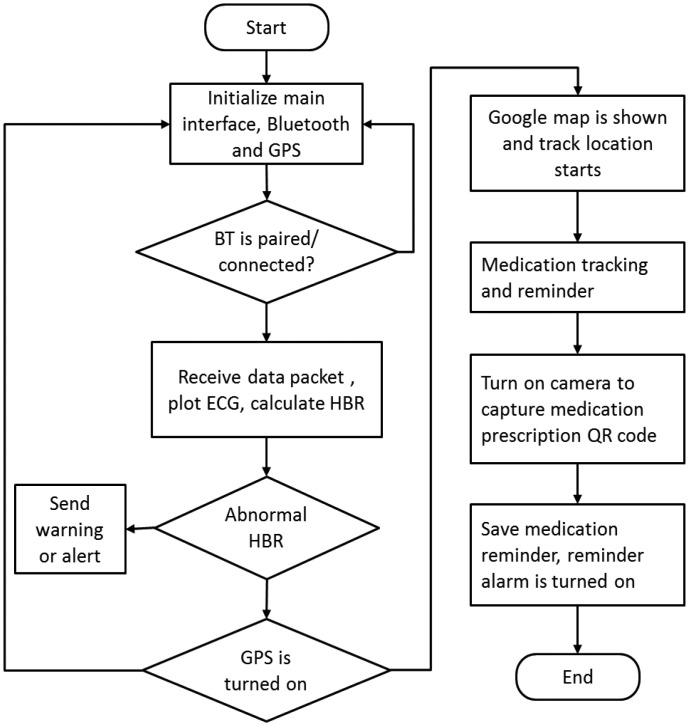
Flowchart of mobile application for health status real-time monitoring and personalized life assistant.

In the case of cardiac arrest, studies have shown that survival falls by 10%–15% without immediate CPR delivery [22]. Thus, the survival rate will become higher. Our system also offers an auxiliary function for medication tracking and reminder. The flowchart of the mobile application for health status real-time monitoring and personalized life assistant functions is shown in Figure 7.

### 3.3. Web Server Cloud Healthcare Service

**Figure 8.**
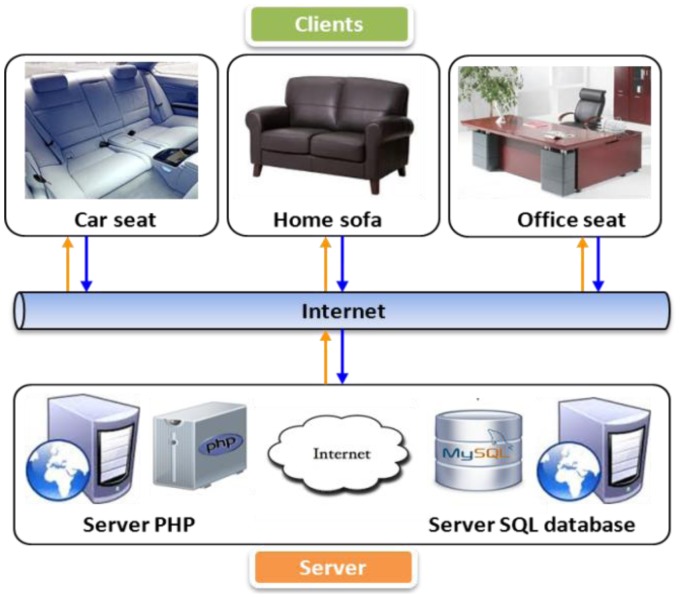
Health monitoring cloud computing architecture where health data from multiple locations are monitored via a cloud server.

Using a Web server healthcare cloud computing system, immediate access to the healthcare tracking system is possible anywhere cellular network coverage is present. The ECG data are displayed in real time on the mobile device. To ensure a seamless and continuous health tracking system, a Web server cloud computing system is implemented into the healthcare service. Figure 8 shows the cloud computing system for health monitoring. The noncontact ECG measurement can be integrated in the car, home, and office, which are the clients of the cloud computing system. The server, which is a combination of PHP scripts and SQL database, will serve and share resources with the clients via the Internet.

#### 3.3.1. Communication between the Mobile and Web Server

**Figure 9.**
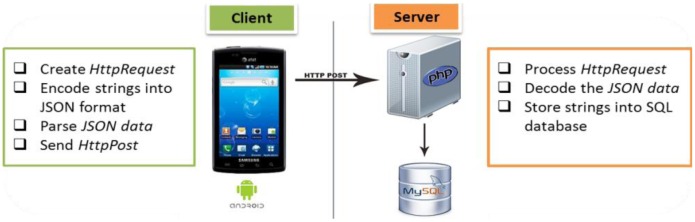
Client-server model. The clients request and receive service from the centralized server.

The relationship between the mobile device and the Web server is referred to as the client-server model. The client is a mobile device or a computer that initiates contact with the server to use a resource. The server is a server system that selectively shares its resources. Communication between the client and the server occurs when the client sends a request and the server returns a response. To communicate, the computers must have a common language so that both the client and the server know what to expect. Figure 9 shows the client-server architecture. The client is an Android mobile device, and the server side consists of a combination of PHP script and MySQL database. The PHP script acts as the connection protocol. Communication occurs when the user clicks an upload button to synchronize the health data. To connect to the PHP script, we use the HTTP protocol from the Android system and the JSON format for data synchronization of the name-value pairs.

#### 3.3.2. Synchronization of Health Data

**Figure 10.**
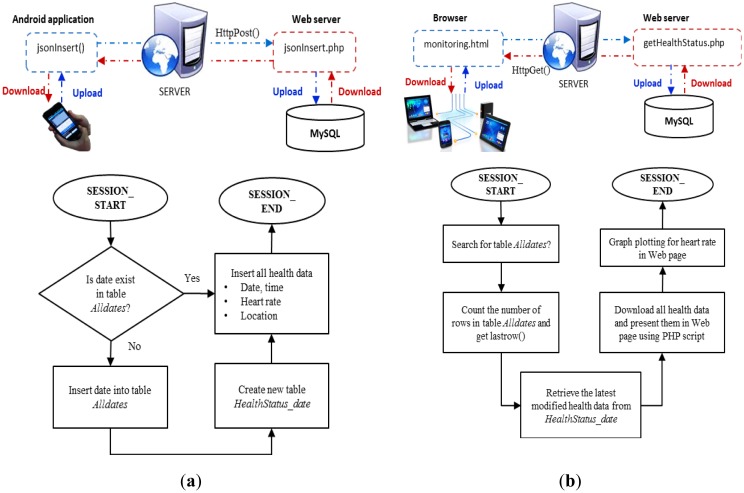
(**a**) Uploading process from the mobile device to the Web server, which involves the creation of a database table and insertion of the health data into the Web server database; (**b**) Downloading process from the Web server to the client side where the clients retrieve the health data from the Web server database.

This feature is intended to synchronize the tables in the database between the Android-based and the Web-based health monitoring systems. To achieve a cloud computing service in the proposed system, synchronization of health data is implemented using HTTP POST and JSON. The algorithms for the uploading and downloading of the health data are shown in Figure 10.

On the client side, which is the Android mobile device application, when the user clicks the upload button, the Android application will call the HttpPost() method defined in HttpClient class, which results in running a PHP script in the Web server. The uploading process from the mobile device to the Web server database is shown in Figure 10a. In this scenario, the date, time, HR, and location are synchronized into the Web server database. First, the exact date and time are inserted in the database with the table name *Alldates*, followed by a new table *HealthStatus_date* based on the newly created date. Then, all the health data, HR, and location are uploaded and stored in the SQL database. At the same time, the Android application calls the jsonInsert() method to build up an array of name/value pairs for the date, table name, date and time in a timestamp format, HR, and location. Then, the PHP script (jsonInsert.php) runs a database query to store the JSON data into the Web server database. The description of the JSON function and the PHP pair is shown in Table 2.

To download health data from the Web server database to the browser, the HTML program calls a PHP script in the Web server where the PHP script retrieves and obtains the health data from the database. The downloading process from the Web server database to the mobile device is shown in Figure 10b. In this process, the PHP script connects to the MySQL database and searches for the table *Alldates*. Then, the number of rows in the table *Alldates* is counted by getting the lastrow() of the table. Next, the PHP script retrieves the latest modified health data from the table *HealthStatus_date* and presents them on the operational Web page. In addition, the retrieved HR is plotted accordingly in a graph using JavaScript.

## 4. Results and Discussion

To ensure integrity and reliability of the proposed system, different experiments are carried out to collect results. A physical real-time monitoring experiment is performed to ensure data transmission from the sensor module to the mobile device. A mobile healthcare assistant application is built to assist the user in his daily life. The health data synchronization and cloud computing system are tested during the experiment to collect all the health data and seamlessly track the health status. A Web server database and an operational Web page are developed for easy health monitoring purposes. Finally, a Web page performance test is performed to ensure a minimal Web page latency and to increase the healthcare service performance.

### 4.1. Real-time Noncontact ECG Monitoring Module

**Figure 11.**
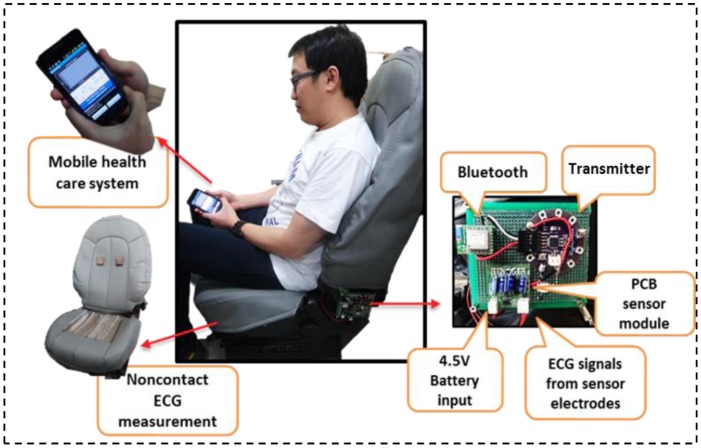
Setup of the ECG real-time monitoring environment.

Our system, which is convenient, noninvasive, cost saving, and based on Web server cloud computing, serves as a smart and seamless intelligent noncontact healthcare monitoring system based on mobile phones and provides life assistance by relaying medication reminders. An implementation of the system is shown in Figure 11. A user wearing a cotton shirt sits on a chair with conductive sensor electrodes installed on the seat. A sensor module, battery supply, transmitter, and Bluetooth module are attached together beside the chair seat, and the user holds the mobile device used for real-time health status monitoring. It is small in size, compact, saves energy, and portable. ECG signals from the user are detected through the cotton shirt and filtered by an electronic circuitry sensor module. The microcontroller and the transmitter relay the data from the sensor module to the mobile phone with an application built specially for real-time health monitoring.

ECG signals can be measured and collected at multiple locations for continuous health tracking. At the same time, users can upload the health data to the Web server for health data cloud computing and synchronization purposes. Thus, the proposed system is very useful for long-term and continuous health monitoring when the user is at home, at the office, or in an automobile. All the health data can be synchronized and collected at the Web server database. An operational Web page is built for easy accessibility and monitoring by doctors, physicians, or family members. Therefore, the main improvement in our proposed non-contact ECG measurement system compared to the already exisiting solutions is that, it provides a seamless and continuous health monitoring for patients or normal users who concern about the health status. This proposed biomedical system with the integration of cloud and mobile service has brought much convenience to users.

### 4.2. Monitoring Service and Personalized Healthcare Assistant

**Figure 12.**
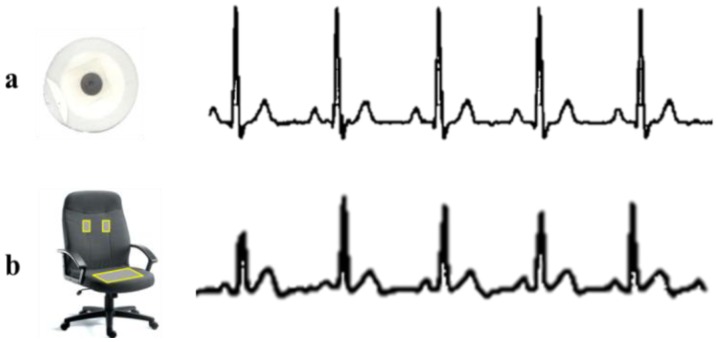
(**a**) ECG signals measured with standard ECG measurement; (**b**) ECG signal obtained using non-contact ECG measurement.

**Figure 13.**
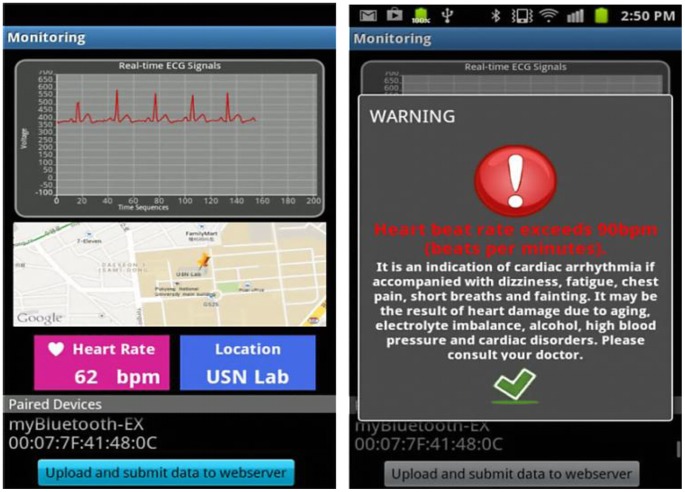
Display interface on a mobile device with ECG plotting, heart beat rate, location, and warning feedback for abnormal heart beat rate.

Figure 12 shows the comparison between the proposed non-contact electrodes ECG and standard ECG measurements. ECG signals are clearly seen in non-contact ECG measurement with only slightly lower QRS amplitudes, which is however not imperative as long as heart beat rate is detected. In addition, Figure 13 shows the mobile interface for the ECG graph plotting that displays the HR, GPS location tracking, and feedback notification for abnormal HRs. The ECG graph is plotted in real time on the mobile device, with the HR updated every 5 s. A Google Maps display based on GPS position information sent from the smart phone is also used to aid medical staff in promptly locating a monitored user who needs further assistance. Meanwhile, the mobile device is intelligent enough to detect an abnormal HR and to feed back an immediate warning to the user on the mobile device to ensure awareness of his or her health condition and to take immediate action to avert death or injury in the event of a possible stroke.

**Figure 14.**
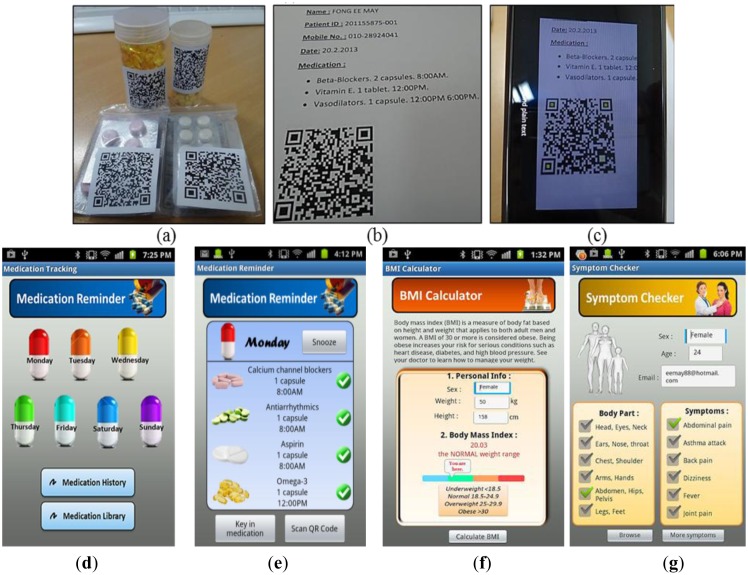
(**a**) QR code on prescribed medicine; (**b**) Personalized QR code medication list; (**c**) QR code scanning on mobile application; (**d**) Main screen for medication reminder; (**e**) Medication alert reminder; (**f**) BMI calculator; (**g**) Symptom checker.

Nowadays, busy lifestyle has resulted in patients or users often forgetting to take their prescriptions. Whereas real-time healthcare monitoring is important, elderly patients may have an additional need to call upon live assistance owing to reduced physical status, discomfort, or decline in faculties such as vision, mobility, or memory. In such circumstances, it may be difficult to memorize medication information, leading to the possibility of mistakes in medication intake. The proposed system therefore uses mobile device-based QR code scanning to digitally store medication intake reminders. Figure 14a shows the QR code for the medicine, whereas Figure 14b shows the personalized QR coding of the prescribed medicines for specific patients. This system allows the elderly or their family members to scan the QR code in the mobile device and save it as a reminder. Figure 14c shows a QR code scanned onto an application using a mobile camera.

After successful scanning and searching, the decoded QR details and information such as medication names, dosages, intake times, and medical purpose can be stored in the mobile application, which will prompt an alarm reminder when it is time to take the medication. Figure 14d shows the main interface for the daily medication intake time and dose reminder information for Monday. To ensure that the user takes his medication on time, a snooze function is added to the proposed system to generate repeated reminders. Figure 14e shows the alert reminder for a specific day. When the reminder is enabled and the prescription is set in the mobile application, the mobile device will periodically issue an alarm to give a kindly reminder. The medication reminder displays the pre-assigned medicine name and intake dosage. Consequently, the user can easily clear the medication alert or view the prescription intake history.

A medical healthcare assistant is important to ensure a healthy lifestyle and healthy body. The application of mobile devices enables users to calculate their BMI for healthy weight monitoring and tracking, which is helpful in preventing obesity and reducing the risk of heart diseases. Figure 14f shows the BMI calculator for BMI monitoring by entering the height and weight of the user. At the same time, the user can check for disease symptoms for early disease diagnosis through the symptom checker function on the mobile device. Figure 14g shows the symptom checker function where the user can browse according to the symptoms or body parts.

### 4.3. Healthcare Cloud Service for Seamless Heath Monitoring

The real-time health data can be uploaded from the mobile device to the Web server database for cloud computing and synchronization. In the proposed seamless noncontact health monitoring system, medical services are extended to multiple locations, multiple users, and multiple devices; the personal details and biomedical data of the users are uploaded onto the Web server monitoring system, making the clinical data and applications readily available to patients, caretakers, and physicians in a community of Internet cloud servers. The relevant health data may also be retrieved from the Web server, enabling intelligent decision making in diagnosis and prognosis by invoking complementary services from the healthcare service network. This process in turn enables continuous and long-term health data monitoring wherever the user is located, as well as allows healthcare professionals to access and evaluate a comprehensive real-time set of patient data at the point of care or anywhere a cellular network coverage is present.

#### 4.3.1. Web Server SQL Database

**Figure 15.**
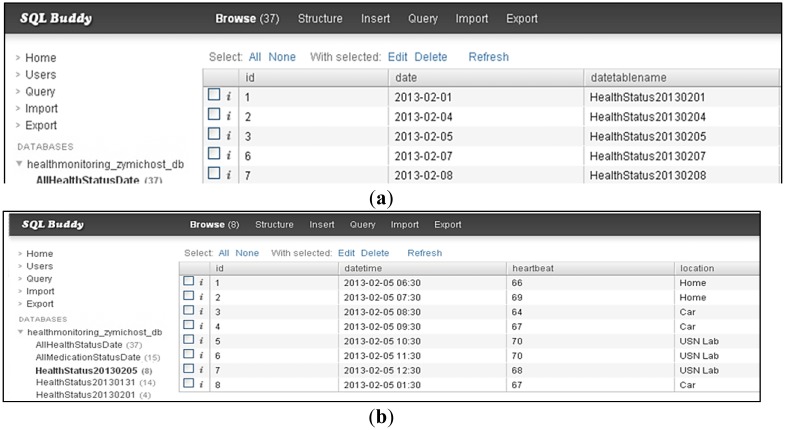

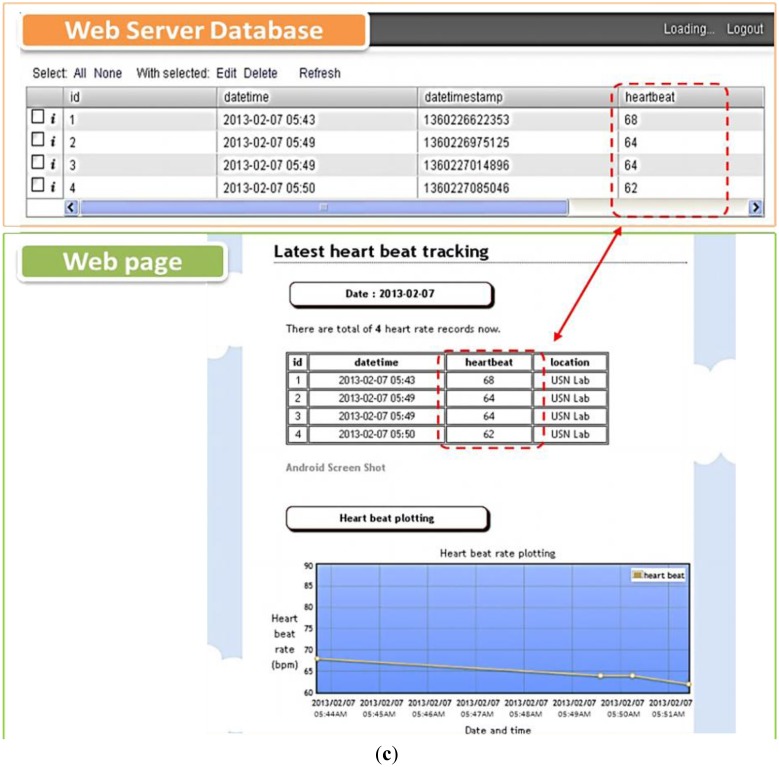
(**a**) Table *AllHealthStatusDate* in a Web server SQL database to store the date and time, and the date table name; (**b**) Table *Datetablename (HealthStatus20130305)* in Web server SQL database to store the date and time, HR, and location; (**c**) Synchronization of health data between Web server database and the Web page. HR is synchronized in a cloud-based architecture system.

To build a cloud computing system, a Web server SQL database is needed to store all the health data at the same database. In our proposed system, SQL Buddy is utilized as a Web cloud computing database. Two main tables are developed in our proposed system, as shown in Figure 15a,b.

Table *AllHealthStatusDate* stores all the dates and date table names, and Table *Datetablename* stores the date and time, HR, and location for that specific date. HR is recorded accordingly in the Web server database, and an HR graph is plotted. Figure 15c shows the HR data in a Web server database retrieved and accordingly displayed on an operational Web page.

#### 4.3.2. Operational Web Page

**Figure 16.**
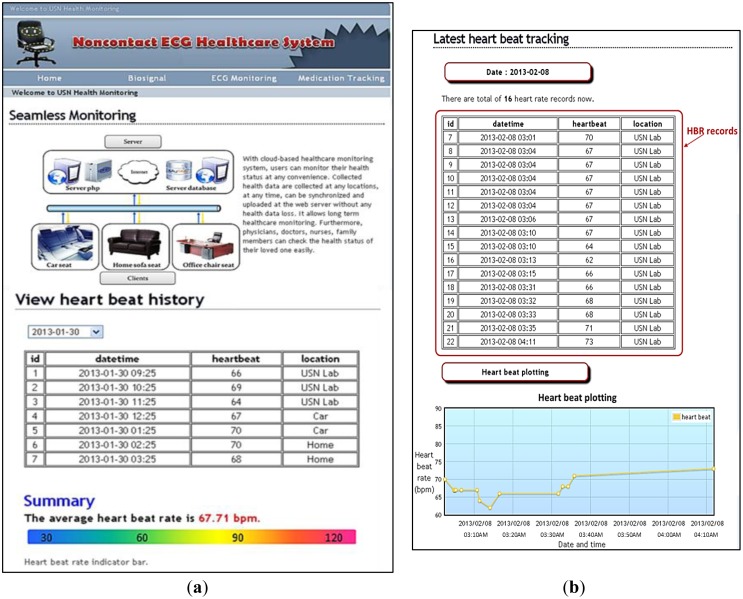
Operational Web page for the noncontact health system for real-time health evaluation wherever a cellular network coverage is present. (**a**) JavaScript dropdown list to view the history and summary of the HR on the Web page; (**b**) HR records and HR plotting on the Web page.

Figure 16 shows the main interface of the proposed system on an operational Web page, which shows the concept of the seamless health monitoring system. The tabs in the operational Web page link the user to the home, biosignal, ECG monitoring, and medication tracking pages. At the same time, the health status history can be tracked by selecting an exact date from the dropdown list on the Web page. Here, easily understandable health details are displayed, and a summary of the average heart beat rate is given. Figure 16a shows an example of the history and summary of the HR tracking. Figure 16b shows the HR records, location, and heart beat rate plotting on the Web page.

### 4.4. Web Server Performance Evaluation

Finally, we analyzed the Web performance latency. Latency is the amount of time it takes for the host server to receive and process a request for a page object. In the case where a Web page contains 100 objects such as images and CSS files, the browser must make 100 individual requests to the site's host server to retrieve these objects. Each of these requests experiences at least 20–30 ms of latency. This latency adds up to 2 or 3 s, which is quite significant because it will slow the Web page down. For obvious reasons, tackling latency is a top priority in the performance industry. Server latency directly affects a site's hardware requirements. For every 20 ms improvement in latency, a linear improvement in page loading times can be realized. Many good reasons can be found for this result: an average page is composed of many small resources, which require many connections, and the TCP performance of each resource is closely tied to RTT. We implemented several changes in the HTTP that substantially reduced latency (individually or together) and reduced the server loading while interoperating with unmodified servers and clients. To improve the Web page performance load time, we minimized the payload by enabling compression, serving scaled images, and minifying the HTML and CSS. In addition, we minimized the delay in the page load by specifying a character set.

**Figure 17.**
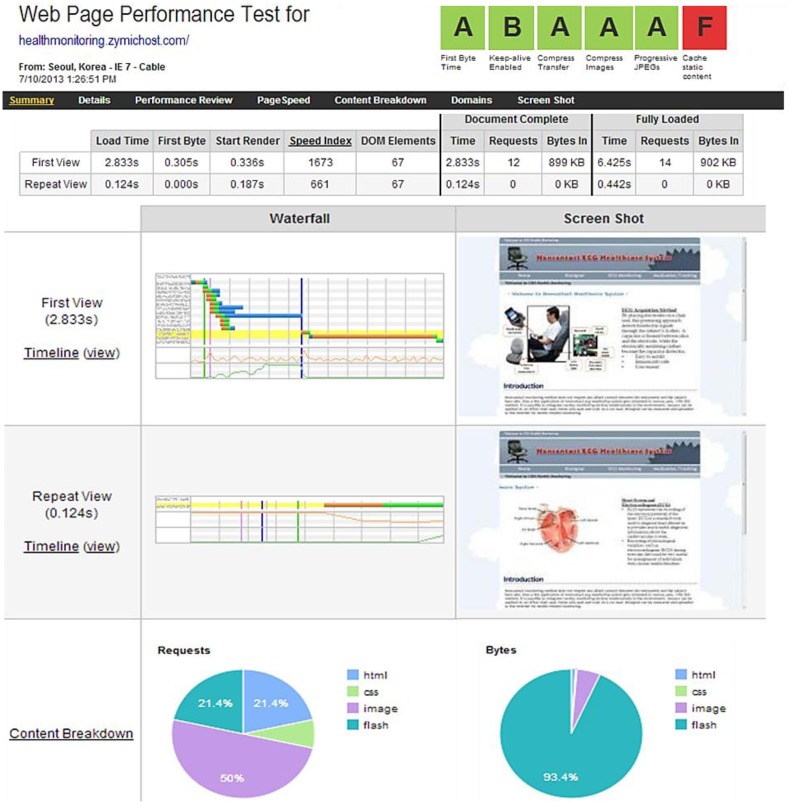
Web page performance evaluation test.

The Web page test is used to measure and analyze the performance of the Web pages. This test measures the elapsed time between the moment a user requests a new page and the moment the page is fully rendered by the browser. Impatience with poor performance is the most common reason why users terminate their visit at Web sites. The best practices cover many of the steps involved in the page loading time, including resolving the DNS names; setting up of the TCP connections; transmitting the HTTP requests; downloading resources; fetching resources from cache, parsing, and executing scripts; and rendering objects on the page. Essentially, the page speed evaluates how well the pages either eliminate these steps altogether, parallelizes them, or shortens the time it takes to complete them. The result statistics for the test run is shown in Figure 17.

For a high-level performance test, the high-level information about the page that is loaded is shown in the data table. To demonstrate performance evaluation, a well-organized overview of the evaluated parameters is shown in Table 3.

**Figure 18.**
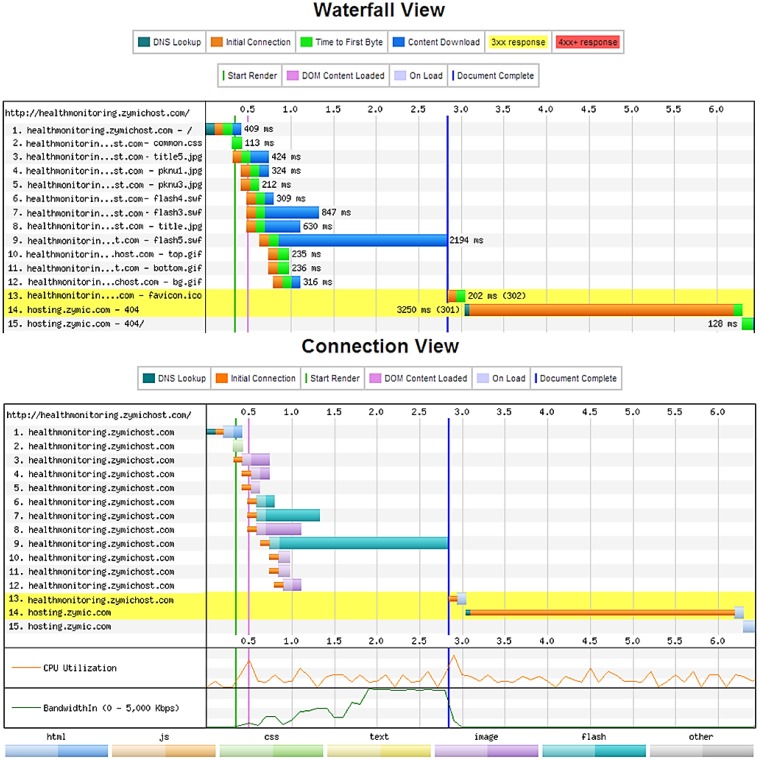
Evaluation in the waterfall and connection views.

A detailed view of the Web page analysis is shown in Figure 18. The waterfall view is used to show the exact timing sequences. Meanwhile, the connection view graph outlines the manner and sequence in which the sites are hit to load various types of data. In the proposed operational Web page system, the First View load time is 2.833 ms while the Repeat View load time is 0.124 ms, which is the optimal performance to load all the health data from the Web server. The Document Complete parameter, which occurs after all the images content (899 KB) have been loaded, requires 2.833 s. Meanwhile, the Fully Loaded parameter, which includes any activity triggered by the JavaScript (902 KB) requires 6.452 s because the JavaScript request and response from the Web server often need higher latency. However, the Web page overall performance is acceptable for the healthcare service and health monitoring system. The performance test demonstrates that minimizing the payload and the delay of the payload substantially reduces the latency while interoperating with unmodified servers and clients. These changes may also help reduce server loading. Thus, a low-cost health monitoring and high-efficiency healthcare cloud computing system is possible.

## 5. Conclusions

A seamless noncontact health monitoring system using a mobile device has been proposed. This system employs a noncontact biomedical signal measurement technique integrated at multiple locations for multiple users. Biomedical data can be acquired unnoticed and digitally processed to identify and recognize the user whose ECG is being measured to ensure that his medical record is stored correctly. Using a mobile device, the user health status is monitored seamlessly and in real time. Simultaneously, the proposed system is intelligent enough to provide feedback to the user when an abnormal HR is detected. To ensure that the health status is tracked continuously and seamlessly, the data are uploaded and synchronized into a Web server healthcare cloud computing system that features an operational Web page specifically developed for health status monitoring. In addition, a medication reminder is implemented on the mobile device to provide personal dosage assistance to the elderly or incapacitated patients. Finally, a Web page performance test was performed to ensure a minimal latency monitoring and efficient health tracking system. Our results demonstrate the validity of the proposed system for use in long-term healthcare monitoring in which medical records can be retrieved by professionals or family members at their convenience using any applicable electronic devices.

## Figures and Tables

**Table 1.** Top-3 causes of death in high-, middle-, and low-income countries.

**High-Income Countries**		

Rank	Diseases	Deaths in millions

1	Coronary heart disease	1.34
2	Stroke and other cerebrovascular diseases	0.77
3	Trachea, bronchus, and lung cancers	0.46

**Middle-Income Countries**		

Rank	Diseases	Deaths in millions

1	Stroke and other cerebrovascular diseases	3.02
2	Coronary heart disease	2.77
3	Chronic obstructive pulmonary disease	1.57

**Low-Income Countries**		

Rank	Diseases	Deaths in millions

1	Coronary heart disease	3.10
2	Lower respiratory infections	2.86
3	HIV/AIDS	2.14

**Table 2.** Description of the JSON function and PHP script pair.

**JSON Function**	**Description**
jsonInsert()	This JSON function and PHP script pair is used to upload the date, table name, date and time in timestamp format, HR, and location into the Web server database.

PHP Script	

jsonInsert.php	

**Table 3.** Performance evaluation parameters.

**Parameters**	**Explanation**	**Tested**
First View	Test done with a browser that has its cache and cookies cleared out and represents what a first-time visitor to the page will experience.	√

Repeat View	Test done immediately after the First View test without clearing out anything.	√

Document Complete	The metrics grouped together under the Document Complete heading are the metrics collected until the browser considered the page loaded.	√

Fully Loaded	The time from the start of the initial navigation up to 2 s of no network activity is experienced after the Document Complete parameter, which usually includes any activity that is triggered by the JavaScript after the main page is loaded.	√

Load Time	The time when the user starts navigating on the page until the Document Complete event is completed.	√

First Byte	The time when the user starts navigating on the page until the first bit of the server response arrives.	√

Start Render	The time from the start of the initial navigation until the first nonwhite content is plotted on the browser display.	√

DOM Elements	The number of DOM elements on the tested page as measured at the end of the test.	√

Requests	The number of requests that must be made by the browser for pieces of content on the page (images, JavaScript, and CSS).	√

Bytes In	The amount of data that the browser has to download to load the page.	√
